# Addressing Health Literacy in Schools in Germany: Concept Analysis of the Mandatory Digital and Media Literacy School Curriculum

**DOI:** 10.3389/fpubh.2021.687389

**Published:** 2021-07-05

**Authors:** Tessa Schulenkorf, Verena Krah, Kevin Dadaczynski, Orkan Okan

**Affiliations:** ^1^Faculty of Educational Science, Interdisciplinary Center for Health Literacy Research, Bielefeld University, Bielefeld, Germany; ^2^Public Health Center Fulda, Fulda University of Applied Sciences, Fulda, Germany; ^3^Centre for Applied Health Science, Leuphana University of Lueneburg, Lüneburg, Germany

**Keywords:** health literacy, school, curriculum, school-aged children, Germany, media literacy

## Abstract

It is generally agreed upon that the development of health literacy should be addressed from an early age onwards in order to empower children to develop their full health potential. Schools can be seen as an ideal venue for strengthening health literacy because they reach almost all school-aged children throughout their school years. The development of health literacy at a young age is a catalyst for healthy development throughout across the life span. Evidence shows that health and education are intertwined with favorable effects for health (e.g., health behavior, knowledge) and education outcomes (e.g., academic achievement). However, health literacy is often not sufficiently integrated into the school curriculum despite its importance to health and education. Integrating health literacy into schools is challenging, as both schools and teachers already face numerous educational requirements that may prevent them from addressing health in the classroom because they perceive it as an additional task. This is why taking a sensitive approach is important, adapted to the needs of schools and highlighting the benefits of health literacy. Installing health literacy in schools succeeds more easily if it can be linked to existing curricular requirements. In this context, curriculum and instruction on media literacy, information literacy, and digital literacy are most promising subjects to include health literacy because these concepts share many commonalities with health literacy and often are already part of the school curriculum. The aim of this article is to (1) analyze a mandatory curriculum on media literacy in the state of North-Rhine-Westphalia in Germany, (2) highlight its intersections with health literacy, and (3) show how it can be used to address health literacy. The state media literacy framework is based on the federal standards for “digital education” developed by the German Conference on Education Ministries und Cultural Affairs (KMK). As education policy and practice is decentralized with sixteen federal states in Germany, each of them has got their own media literacy framework, or they are currently developing it. This curriculum analysis may serve as a methodological blueprint for educationalists, teachers, and policy-maker elsewhere in order to include health literacy into existing curricula both health and non-health. It may help to integrate health literacy into schools when combined with existing curricula.

## Introduction

In a rather conservative approach to the concept, health literacy merely describes a person's ability to deal with his or her health-related functional literacy skills and navigate the health care system (1). However, the health literacy concept has evolved into a modern key competence of health promotion and prevention equally focusing on finding, understanding, and communicating health information, making critical judgments about health claims, and empowering individuals to make informed health decisions, practice healthier behaviors, and modifying the personal determinants of health (2, 3). In this context, many models and definitions appeared over the past decades for adults (4, 5) as well as for children and adolescents (6, 7). A common and often quoted definition of health literacy, also representing the main commonalities across available definitions, is the one presented by Sørensen et al. (4):

“Health literacy is linked to literacy and entails people's knowledge, motivation and competences to access, understand, appraise, and apply health information in order to make judgments and take decisions in everyday life concerning healthcare, disease prevention and health promotion to maintain or improve quality of life during the life course”.

The core of this definition focuses certain action areas related to competencies to deal with health information, namely (i) accessing, (ii) understanding, (iii) appraising, and (iv) applying. In this sense, health literacy can also be conceptualized as information literacy with regard to health topics (8, 9). New communication channels have emerged with increasing digitalization. This means that information is not only sought in analog form, but rather and especially in digital media such as social media (10). The concept of digital health literacy conveys this understanding of health literacy specifically to digital contexts and environments (11). Children and adolescents seek, adopt, and produce digital information on the internet partly also on social media (10). Thus, there is a close conceptual relationship between health literacy on the one hand and media, information, and digital literacy on the other (12–14). They essentially share the competencies to deal with (health) information. Hence, strengthening health literacy fits well with strengthening these literacies.

Emerging evidence suggests that fostering health literacy as early in life as possible is preferable since it is associated with better proximal and distal health and social outcomes (15–17). Focusing on early life helps children and adolescents to grow into health literate adults, who have learned and internalized the skills, competencies, knowledge, attitudes, and beliefs to handle health information on an individual level (18–21). This is why it is important that health literacy is embedded into a socioecological approach, encompassing behavioral and structural components (22). Schools have long been identified as strong venues for health promotion and health education (19, 23–25) since schools can reach almost all school-aged children regardless of their social, cultural, or economic background. This is particularly important because studies indicate a social gradient in the incidence of low health literacy in children and adolescents (26–28). School-based interventions aiming at promoting health literacy can contribute to reduce health inequalities (20). Low health literacy in adolescence is associated with harmful and risky health behavior in adulthood and poorer health in general (20, 29). It is also relevant that students ^1^ develop skills to learn about (their own) health because much of the lexical knowledge will be insignificant when they are adults (24). In the Shanghai Declaration on promoting health in the 2030 Agenda for Sustainable Development (30), the World Health Organization (WHO) also calls for the early promotion of health literacy in the education system:

“Health literacy is founded on inclusive and equitable access to quality education and lifelong learning. It must be an integral part of the skills, and competencies developed over a lifetime, first and foremost through the school curriculum”.

However, health literacy goes beyond an individualistic and behavioral approach and includes the structural and environmental levels (22, 31). In the context of education, health literacy can be addressed at the organizational level of schools in order to improve structural factors and lower barriers that hinder appropriate action on health literacy (20). Related to this, the health literacy of teachers, principals, and school staff is just as important as the health literacy of their students, especially because studies have shown that teachers' and principals' health literacy are associated with the implementation status of health promotion in schools (20, 32–34). In the best-case scenario, school-based interventions to promote health literacy should address both, the individual and organizational level as part of a holistic Health Promoting School (HPS) approach (20, 21). However, HPS is not available in all countries but similar concepts or whole of school approaches could be used as well to address and implement health literacy (20, 21).

Health literacy is known to be the outcome of health education at schools (1, 20). However, in Germany health education is not part of the mandatory school curriculum, which makes it difficult to address health literacy in school. Health, including health promotion and prevention, is often implemented in an unsystematic way, e.g., through school projects limited in time and scope (35). Often, health interventions introduced to the school context fail to be successfully incorporated into the curriculum and thus are not sustainable. One reason for this is rooted in teachers' and school professionals' perceived lack of fit between the subject matter of health and the core mandate of education, which in turn has to be understood as a key barrier in its own right, impeding uptake and sustainable implementation of health topics in schools (36). Another factor that hinders the uptake of health in schools is an overcrowded curriculum, missing time and professional resources, and, partly, the lack of competencies and knowledge of the school staff (18–20). Altogether, this makes it difficult to systematically address health in schools. It will need approaches that overcome these barriers, especially when aiming at strengthening health literacy in schools. In the German federal state of North Rhine-Westphalia (NRW), like in most other German states, there are specific non-mandatory state programs to address school health promotion and education. In NRW, the state program ‘health and education’ includes the promotion of health literacy as one of the objectives to be accomplished by 2022 (37). By strengthening health literacy, it is supposed to improve overall health outcomes and educational opportunities of students in the long term. However, the state program is not mandatory, and many schools do not participate in the program, which is why health literacy has not been included systematically and across schools to the curriculum. For it to be included to the curriculum or classroom activities, it will be important to align health literacy with the core tasks and goals of schools. The health literacy learning activities must be easily adaptable to daily school routines without additional efforts for teachers and no need for additional resources for the schools (20, 21).

Since there is no mandatory health education in schools in Germany with health literacy as the desired outcome, different approaches and entry points are needed. In order to identify such entry points for health literacy within the school curriculum and possible intersections with the core school tasks and concepts, existing school curricula should be analyzed first. This way, subject areas, which can be easily linked with health literacy, can be identified. On the national level, a framework for digital education and digital literacy in schools (38) has been introduced recently, which consists six dimensions how to address ‘education in a digital world’ in schools. This framework has been adapted into the school media literacy framework on the state level. The new framework was designed as a cross cutting theme that can be used across subjects or other cross-sectional school topics and issues. The framework comprises digital literacy, media literacy, and information literacy, which share many similarities and commonalities with health literacy as highlighted earlier (9, 12, 13). Therefore, the new media literacy framework seems to be such an entry point and hence a promising opportunity for addressing health literacy in schools. Especially since (digital) media-related competencies are becoming more important as a result of increasing digitalization and the digital transformation of society (39, 40). In contrast to existing curricula, media literacy frameworks are still fairly new in Germany and will only become compulsory in summer 2021 (41), although there is a long tradition of media education and pedagogy in schools dating back to the 1990s.

The aim of this article is to discuss how health literacy could be introduced to schools, taking into consideration (1) that no additional efforts are required but (2) it would be integrated into existing teaching and learning frameworks. First, the German digital education framework and the new mandatory curriculum on media literacy of North-Rhine-Westphalia will be presented and the underlying concepts will be analyzed. Second, the intersections with health literacy will be highlighted. In addition, this approach to curriculum analysis can be seen as a methodological blueprint that can be easily adapted to educational systems of other countries so that they could integrate health literacy in their curricula without the need of any extra resources and in alignment with their educational goals.

## Concept Analysis of National Pedagogical Framework

To assess state or federal school standards in Germany that share similarities and commonalities with health literacy, we have reviewed available documents of the education sector in online databases (both federal and state-level) as well as on the website of the KMK. We focused the concept analysis on:

1. identifying governmental frameworks and recommendations for topic-based or cross-cutting issues,
2. analyzing available concepts that provide possible interfaces to address health literacy.

In the available school curricula various subjects, topics, and competency frameworks were found to intersect with the core action areas addressed by health literacy. After analyzing the findings, the study team decided to use the national digital education and digital literacy framework and the state-level media literacy frameworks because they show the best fit to health literacy.

Through their recently introduced strategic concept ‘Education in the Digital World’ (In German: “Bildung in der digitalen Welt”) (38), the KMK presented new national standards for addressing digital education and digital literacy in the school. This framework was developed in collaboration with all 16 German federal states and involved stakeholders from science, unions, associations, and different levels of administration and practice. Three strategies make up one federal act based upon three key documents: (i) The Education campaign for the digital knowledge society' (“Bildungsoffensive für die digitale Wissensgesellschaft” in German) (42), (ii) The ‘Digital Pact Schools’ (“DigitalPakt Schule” in German) (43) and (iii) The concept ‘Education in the Digital World’. Together they also aim at preparing students, teachers, schools, and the whole education sector for the digital transformation of society and challenges associated with digital changes in all areas of life, including education. Part of the strategy is to establish a digital infrastructure in schools (including computers and internet access) and to create new training and education opportunities for in-service and pre-service teachers and educational staff. On the state-level, this new strategy includes a mandatory educational framework to foster digital literacy in schools, which will be implemented nation-wide, starting in 2021. Based on the digital literacy approach, the states have defined their own teaching and learning goals for promoting digital media skills in schools. Therefore, in the state-level education systems the national digital education and digital literacy framework is translated into a ‘media literacy framework’, and will also be a mandatory curriculum item in teacher training at University levels in all federal states, also starting in 2021.

The national framework outlines six action areas (also called ‘competence areas’) to ensure that all children have been taught media and digital skills in schools by 2026. These six areas of “Education in the Digital World” are briefly described below (Table 1).

**Table 1:** Strategy and competence framework for digital education and literacy by the KMK (38).

**Competence areas for digital education and digital literacy** (38)		
1.	Searching, processing, storing	This includes searching and filtering sources and information in various digital environments, evaluating and assessing these information and sources, and storing and retrieving various information and data.
2.	Communicating and cooperating	This means interacting with the help of digital communication technologies, sharing data and information, working with different digital tools, knowing and adhering to rules of conduct and actively participating in society.
3.	Producing and presenting	Summarized by developing and producing, processing and integrating various contents and observing legal requirements.
4.	Protect and act safely	This comprises acting safely in the digital environment by considering risks and dangers, protecting personal data and privacy, protecting health by using digital technologies in a health-conscious way and protecting nature and the environment.
5.	Problem solving and acting	This encompasses solving technical problems, using digital tools as needed, identifying own deficits and searching for solutions, recognizing and formulating algorithms.
6.	Analyzing und reflecting	This contains the analysis and evaluation of media offers including the intentions and effects of information provision and the comprehensive understanding and reflection of media in the digital world, including the chances and advantages, but also the risks and disadvantages.

This concept is based on earlier life-skills and digital literacy approaches and defines the areas of competence in which students should learn digital and media skills at school and classroom levels. Like many school topics in Germany, this strategy is meant to be a cross-cutting issue that should be addressed across subjects and not only in specific school subjects such as math, science, or language. This framework represents both a guidance and action plan to further develop digital education in Germany.

Due to the federal states' sovereignty in Germany, each state is responsible for defining its own strategy for digital education. Many states have already adapted the strategy for education in the digital world and have embedded it in their own frameworks, which historically are often rooted in media education and media literacy (sometimes also called ‘media pedagogy’). On the state level in North Rhine-Westphalia, the national model was adapted into the Media Literacy Framework (“Medienkompetenzrahmen NRW” in German) (41) using slightly modified titles and content for the dimensions compared to the original model (Table 2).

**Table 2:** Media literacy framework North-Rhine Westphalia, Germany (41).

**Media literacy framework NRW** **(****41****)**		
1.	Operating and applying	Describes the technical ability to use media sensibly and is the prerequisite for all active and passive media use.
2.	Informing and researching	Includes the sensible and targeted selection of sources as well as the critical evaluation and use of information.
3.	Communicating and cooperating	Accord to rules for secure and targeted communication and to use media responsibly for cooperation.
4.	Producing and presenting	To know about media design possibilities and to use them creatively in the planning and realization of a media product.
5.	Analyzing and reflecting	Is to be understood in two different ways: On the one hand, this competence comprises knowledge of the diversity of media, on the other hand, it amounts to the critical examination of media offers and one's own media behavior. The goal of reflection is to arrive at a self-determined and self-regulated use of media.
6.	Problem solving and modeling	Amounts to a basic informatics education as an elementary part of the educational system. In addition to strategies for problem solving, basic programming skills are taught and the influence of algorithms and the impact of process automation in the digital world are reflected.

This framework is to be understood as rather generic and covers all areas relevant to educating students to become digitally literate. In Germany, educational efforts are meant to be inclusive and integrative. They not only focus on narrow skill areas but holistically address a concept in a broader sense, which is why it makes sense to think of health literacy holistically, too. Ideally, the students learn all necessary competencies, the relevant factual and practical knowledge etc. Then, health literacy can be linked to other relevant dimensions of the framework that go beyond the health literacy definition by Sørensen, et al. (4) presented earlier. If health literacy is understood (as part of) a process of developing this competencies rather than a cognitive concept to be transferred to the student, students will automatically learn more than just health-related information literacy skills while achieving the aims of the core health literacy action areas. They must learn techniques of media use, communication, problem-solving skills, and many more as outlined in Table 2. Therefore, embedding health literacy into this framework departs from the definition presented earlier as it interlinks health literacy with various competence areas. In addition, it significantly contributes to a holistic conceptualization of health literacy as expected by the education sector. Doing so has several benefits, as the curriculum

1. addresses the core action areas of health literacy (the information literacy skills),
2. allows linking health literacy to the context of digital media and (communication) technology environments and associated requirements,
3. includes components to address social, emotional, ethical, and psychological development to support the learning of self-regulation, identity creation, and opinion forming in context of health literacy, and
4. permits the linking of necessary health literacy skills with other critical skills that are needed when aiming at finding, understanding, evaluating, and using information to promote health.

In the following we present the six core dimensions in more detail, including the 24 sub-dimensions of the framework, and adapt them to health literacy (Table 3). The model provides a curriculum and associated learning achievement goals across age-grades, including primary, secondary and upper-secondary students. While the dimensions are the same for all age groups, the age-adapted goals differ in their complexity and depth, increasing with age and children's cognitive and social development stages. For the purpose of our analysis, we will focus on the model itself rather than on specific age groups. The exemplary exercises in the third column are based on the learning and teaching examples given in the original framework. They are thought to demonstrate various possibilities and help to imagine how to operationalize and implement the promotion of health literacy in the school setting.

**Table 3:** Main dimensions and competence areas of the media literacy framework in North-Rhine Westphalia, Germany (41), and pertinent exemplary health literacy exercises.

**Main dimension**	**Competence area**	**Exemplary exercises and learning goals regarding health literacy in class**
1. Operating and applying	1.1 Media equipment (hardware)	Using a mobile phone and a tablet to search for health information.
	1.2 Digital tools	Using different tools or web-based applications (e.g., PowerPoint) to filter, summarize and creatively represent health information.
	1.3 Data organization	Securely storing, retrieving and accessing health information and data from multiple locations.
	1.4 Data protection and information security	Ensuring data protection, privacy and information security of online health information and storing data on a hardware.
2. Informing and researching	2.1 Information seeking	Defining a search topic, search strategies, and terms related to health needed to search for information.
	2.2 Analyzing information	Understanding, filtering, structuring, and preparing health information and being able to grasp and describe their meaning.
	2.3 Evaluating information	Critically evaluating the quality of health information and identifying strategies and intentions behind health information, sources, and information providers, and fact checking their reliability against other sources.
	2.4 Critical information review and use	Recognizing inappropriate health media content and estimate its legal base and the underlying social norms; knowing youth and consumer protection and using health-related support and assistance structures.
3. Communicating and cooperating	3.1 Communication and cooperation processes	Communicating and collaborating in groups of students through digital tools to share search health information results with the class.
	3.2 Communication and cooperation rules	Knowing and understanding the rules of (digital) health-related communication and using those when interacting with others.
	3.3 Communication and cooperation in the society	Creating health-related (digital) communication processes in the sense of participating in society and understanding ethical principles with regard to social norms and applying them on the internet.
	3.4 Cyber violence and cyber crime	Knowing the risks and effects of cyber violence and knowing how to deal with them when using the internet for health issues.
4. Producing and presenting	4.1 Media production and presentation	Planning, designing and presenting search results regarding health information, preparing them to share in class.
	4.2 Design tools	Knowing different design elements of media products, e.g., audio and video, radio plays, explanatory films or animation, and applying them in a reflective manner for presenting health information to others.
	4.3 Documentation of sources	Providing all sources of the health information and data used at the end of a PowerPoint presentation, which allows other to check the sources.
	4.4 Legal basis	Understanding and applying copyrights and rights of use when using images or illustrations during the creation and presentation of health-related content.
5. Analyzing and reflecting	5.1 Media analysis	Comparing a scientific article in a journal with a newspaper article in a daily magazine with respect to their health information.
	5.2 Opinion forming	Analyzing the spread of fitness and nutrition trends and commercial intentions on social networks (such as Instagram, YouTube, Twitter, TikTok) and understanding the power of and how influencers can form opinions as part of their job.
	5.3 Identity creation	Understanding how social networks disseminate health topics that can influence perceptions of reality and using this insight for their own identity building, e.g., through reflecting the difference between virtual and real world.
	5.4 Self-regulated media use	Being able to critically evaluate the effects of the media and to use them for health-related topics in a responsible manner.
6. Problem solving and modeling	6.1 Principles of the digital world	Comparing different search machines on the internet (e.g., DuckDuck, Google, Ecosia) and different hardware (e.g., mobile phone and tablet) and analyzing the results of the gathered health information.
	6.2 Recognizing algorithms	Recognizing how health information results and medicine advertising on the internet change when certain health keywords are searched for on commercial sites.
	6.3 Modeling and programming	Programming a bot with a construction-app so that they may be able to bypass algorithms on social media.
	6.4 Importance of algorithms	Analyzing the influence of algorithms on the digitized society and the effects of automation, e.g., when dealing with a research for health information.

There are many entry points and intersections for addressing various actions connected to the handling of information and knowledge relevant to health and well-being. Especially the second dimension, “Informing and researching” and its sub-dimensions 2.1, “Information seeking”, 2.2, “Analyzing information”, 2.3, “Evaluating information”, and 2.4, “Critical information review and use”, can function as interfaces to strengthen health literacy. Here, this understanding of health literacy can be applied seamlessly and there is no need to alter it in terms of the dimensional specifications.

To practice and strengthen health literacy and digital health literacy skills, other dimensions of the media literacy framework may be of use, too. When students search for various health information, e.g., on a smartphone, computer, or tablet, effectively, they “operate and apply” digital media (Dimension 1). In addition, group and tandem work, which is regularly practiced in schools, automatically addresses the third dimension, “Communicating and cooperating”, and also includes the social component which is important to health literacy. Dimension 4, “Producing and presenting”, is addressed when the search results need to be prepared for class presentations. Dimension 5, “Analyzing and reflecting” of media, is closely related to the health literacy action areas “critical thinking” and “appraisal of health information”. If students frequently search for health-related content on the internet, they will need the skills to reflect and analyze the content they access and distinguish accurate from false and misleading information. Dimension 6, “Problem solving and modeling”, is trained when students encounter digital problems during their research and have to solve them. This is also closely related to applying information. In the long run, they are trained in data literacy and in their understanding and use of algorithms to detect patterns in information flow and communication. This understanding can then be used to identify digital principles and use them consciously. The fifth and the sixth dimensions also share intersections with evaluating information and critical thinking about health claims. While the competencies and action areas presented here seem to go beyond the common health literacy concept, reviews on health literacy concepts and measurement tools for children and adolescents show that most of the competencies and action areas are in fact used in many of the available concepts and tools (7, 15, 44, 45).

## Discussion on the Practical Implications

### Opportunities

Based on a socioecological understanding of health as presented by Whitehead and Dahlgren (46), there is a wide array of factors determining health development of students. The peril of addictive substance abuse (such as nicotine, cannabis, alcohol, or synthetic drugs), the influence of fitness trends and nutritional advice on social media, dealing with changes in the body, and being confronted with body ideals in print media and in the digital realm are important topics, affecting children and young people, and their peer groups. Many of these health-related topics are communicated on media channels, especially the internet and social media. Combining media and health skills would help students navigating these contents and environments. It would also equip them with the ability to critically evaluate online health messages and claims as well as their digital sources and suppliers. Educating and teaching students the competencies and facilitating the development of attitudes and beliefs in relation to media is necessary because today‘s generation grows up being socialized with social media as national (47, 48) and international studies (49) show. A German study from 2019 with 140 students from different types of schools in the 9th grade showed that their self-perceived skills to critically think about and deal with online sources and information tends to be overestimated compared to their actual performance (50). When selecting health information from a Google search, 19.2% tended to take a marketing website that advertises pharmacy products to be credible. In addition, 18.6% indicated that they never or rarely cross-check online information with other sources. The study also showed that students tended to take the number of followers as an indicator of the validity of information within a raffle on social media (50). Another German study revealed that adolescents frequently reported difficulties in searching for, evaluating, and assessing the personal relevance of digital health information (51). Despite growing up with digital and social media, it seems that adolescents are still inexperienced regarding the critical assessment and handling of digital health information and, above all, fail to make accurate judgments about health claims and messages. These issues are important to address through health literacy in schools.

### Challenges

Consequently, several challenges have to be mentioned, and they have to be considered in present educational efforts and prospectively. Addressing health and health promotion, including health literacy, in the school setting requires professional knowledge about the health-related needs of a heterogenous group of students with different cultural norms and social beliefs. Educator's health literacy is an enabling factor for the development of adequate students' health literacy, which is why teachers' and principals' health literacy should be equally strengthened in order to improve the quality of school health education (20, 21, 32). Teachers are required to be familiar with digital tools and teaching in, about, and by using virtual environments in order to motivate students to learn about and engage with health literacy. Accordingly, media skills and building up the confidence to use digital education tools in classroom should be part of the teacher training curriculum, not only at universities but also in practical trainings of pre-service teachers at schools. In-service teachers will need vocational training and education to become familiar with digital education and literacy teaching and learning to be able to pass on the knowledge, competencies, and values to their students, including various instructional and didactical methods.

In addition, materials, programs, and interventions must be available for teachers and schools to address health literacy. Presently, there are few teaching materials and their didactic implementation often is difficult. Therefore, interventions and didactic materials are much needed.

## Conclusion

To prepare and facilitate a better implementation of health literacy in schools, it is highly important to “speak the language” of education and understand the needs of teachers, schools, and the education sector (18). A most promising approach to include health literacy as a learning goal to schools is to identify entry points in existing school curricula and educational policies, which can be done by analyzing the national and/or local school curriculum and seek for concepts, topics and themes that share commonalities with health literacy. With the German strategic framework for digital education and the associated media literacy frameworks on the state levels, we identified such entry points, which may exist in other countries as well. They provide a foundation for integrating health literacy and developing educational interventions to strengthen health literacy in school, and they can be interlinked with further cross-cutting topics such as health promotion, physical activity, or mental health. In addition, as this framework is also meant to be addressed in University curricula for teacher training and education, teachers would be able to use health literacy within a framework they are familiar with already. When using these frameworks for addressing health literacy, teachers would not have to make an extra effort. Analyzing existing curricula, identifying entry points, and adapting the frameworks accordingly could be a methodological blueprint for other countries to analyse their curricula and address health literacy through digital, media, and information literacy, other literacy frameworks or even whole new topics that allow to incorporate health literacy.

## Data Availability Statement

The original contributions presented in the study are included in the article/supplementary material, further inquiries can be directed to the corresponding author.

## Author Contributions

TS, VK, KD, and OO wrote sections of the manuscript. All authors contributed to manuscript revision, read, and approved the submitted version.

## Conflict of Interest

The authors declare that the research was conducted in the absence of any commercial or financial relationships that could be construed as a potential conflict of interest.

## Footnotes

^1^We use “student” to refer to all school-aged children.
